# Water Hyacinth's Extent and Its Implication on Water Quality in Lake Victoria, Uganda

**DOI:** 10.1155/2023/4947272

**Published:** 2023-03-31

**Authors:** Hussein Kiyemba, Bernard Barasa, Joyfred Asaba, Paul Makoba Gudoyi, Gertrude Akello

**Affiliations:** Department of Geography, Kyambogo University, P.O. Box 1, Kyambogo, Uganda

## Abstract

Water hyacinth (*Eichhornia crassipes*) degrades and obstructs the integrity of freshwater ecosystems. However, little attention has been paid to monitoring water hyacinth's spatial extent, its determinants, and its effects on water quality in Lake Victoria, Uganda. The specific objectives of this paper are to (i) assess the spatial extent and distribution of water hyacinth; (ii) examine the determinants of water hyacinth distribution, and (iii) assess its impact on water quality. High-resolution satellite images (2016–2019) were obtained and used to monitor the spatial extent of the water hyacinth, a household survey was conducted to examine the determinants of the water hyacinth's extent and patterns while water samples were drawn and analysed for physicochemical properties. Results show that the coverage and distribution of water hyacinth varied over space and time. Water hyacinth coverage primarily increased with a decrease in water surface area. The perceived factors that triggered the water hyacinth spread included the morphology of the Bay, effluent discharge, strong winds, speed of water current, water-level changes, ferry navigation, and construction activities at the shore. Water parameters significantly impacted by hyacinth were pH, TP, BOD, COD, DO, turbidity, and transparency. This study recommends the strict development and implementation of integrated weed control measures, catchment management plans, and point and nonpoint pollution source control.

## 1. Introduction

Water is an irreplaceable and indispensable natural resource, vital for life on earth, economic development, and human well-being [1, 2]. Although 71% of the earth's surface is water, not all the water is accessible and suitable for all uses. Useable water is meagerly available in fresh water streams and inland lake systems. However, even in these conditions, water quality is continuously deteriorating, thus raising sustainability concerns [3]. The water quality deterioration due to pollution is currently the principal challenge to water resource management [4–6]. Water is polluted if it cannot serve a particular purpose resulting from processes that alter its physical, chemical, and biological constituents as it moves through the various spheres of the hydrological cycle [7].

Physical, chemical, and biological constituents define water quality and its suitability for various uses [8]. These components are affected by several factors, including storm runoff, nitrification from decayed matter, water hyacinth, toxic and hazardous substances, oils, grease, litter, rubbish, and land use such as industrialization, farming, mining, and forestry activities, which significantly contribute to water quality degradation [9–11]. Land uses either increase the concentration of nutrients or suspended materials (as is the case with agricultural land use) or increase the supply of heavy metals and toxic substances in water (like is the case with industrial activities) [12].

Among the biological sources of water quality deterioration is the eutrophication from aquatic plants [3] such as the water hyacinth (*Eichhornia crassipes*), a free-floating perennial monocotyledonous plant belonging to the family Pontederiaceae. This hydrophyte possesses the potential to alter water nutrient cycles and impact aquatic life [13, 14]. The water hyacinth degrades and damages freshwater systems, compromising water quality and threatening the quality of life [15]. Aquatic weeds represent one of the growing challenges for biosecurity and water resource management worldwide [16]. The costs associated with the management of this aggressive waterweed are enormous. In Africa, the damages are estimated at an annual cost of $100 million [17].

Risks related to aquatic weeds have been on the rise due to climate change effects and increased nutrient enrichment, as well as other organic and inorganic pollutants from various anthropogenic activities [18]. Despite threats posed by these weeds and their relative increase in spatial coverage, there have been minimal monitoring and management efforts. Besides, their spatial distribution and configuration remain poorly quantified and less understood particularly in less developed economies [19, 20]. Timely detection and up-to-date information regarding water hyacinth distribution are crucial in understanding its spatial configuration and propagation rates [21]. Monitoring and mapping the spatial configuration of water hyacinths are necessary to provide essential information for proper mitigation and control and ensure the continued provision of goods and services by the water bodies under such threats [19]. With the recent developments in remote sensing science and geographical information technologies, it is possible to undertake such resource assessment and monitoring tasks with ease. These technologies enhance our ability to acquire spatial data and study and map landscape features such as vegetation for timely inventory and assessment of such resources [22]. Satellite data can capture the spatial and temporal distribution of aquatic macrophytes in a timely and cost-effective approach [23, 24].

Previous studies in the Lake Victoria basin have focused on urban eutrophication and its spurring conditions, the socio-economic impact of water quality deterioration, and the impact of effluent discharge on water quality [25–29]. However, little attention has been paid to monitoring the spatial extent of water hyacinths, their determinants, and their effects on water quality in Lake Victoria. Moreover, studies detecting the spatial distribution and configuration of water hyacinth involving the use of Geographical Information Systems (GIS) and relatively high-resolution remote sensing data such as Sentinel-2 imagery are scanty in the region. With the help of remote sensing and GIS tools, essential information for proper mitigation and control of the waterweed can be acquired and thus reduce contamination levels of the water in the lakes. Therefore, the specific objectives of this paper are to (i) assess the spatial extent and distribution of water hyacinth; (ii) examine the determinants of water hyacinth distribution, and (iii) assess its impact on water quality in Lake Victoria, Uganda. It is therefore imperative to map the distribution and assess the effect of this alien aquatic plant species on the quality of water in the lake, so that appropriate control and management measures are implemented to keep contamination at unproblematic levels.

## 2. Materials and Methods

### 2.1. Description of the Study Area

**Figure 1:**
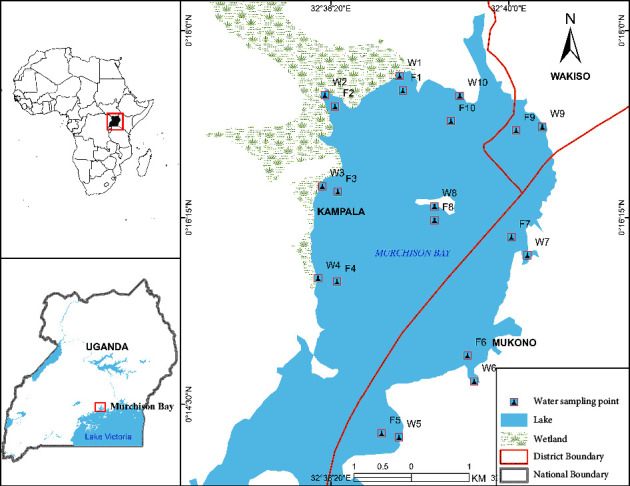
Water sampling locations in the Murchison Bay, Lake Victoria (W1∼W10 = water hyacinth-infested sampling areas while F1∼F10 = open lake environment sampling areas).

This study was conducted in the Murchison Bay which is part of Lake Victoria. It covers parts of Kampala city and Mukono and Wakiso districts in Central Uganda. The Bay stretches between latitudes 0°13′5″ N–0°18′67″ N and longitudes 32°36′59″ E–32″40′27″ E, forming an extension of Lake Victoria (Figure 1). Lake Victoria is located in the south east of Kampala city, lying between latitudes 0°10′00″ N–0°30′00″ N and longitudes 32°35′00″E−32°50′00″ E with an average elevation of 1,224 meters above sea level. Temperatures around the Bay range from 25 to 32°C while winds are around 6.9 km/h north [30, 31].

The Murchison Bay covers an area of about 62 km^2^ but with a catchment area of approximately 282 km^2^. The depth of the Murchison Bay in 2004 was 7 meters, but by 2008, it had dropped by 1½ meters [26, 31]. The Bay is further split into inner and outer sections as their characteristics differ tremendously. The inner Murchison Bay is a semienclosed small water body with an area of 25 km^2^ and a length of 5.6 km off the main lake section. This section is relatively shallow with an average depth of 3.2 m but deep towards the main lake area with a convoluted shoreline and narrow at the exit to the outer Murchison Bay. These facilitate the mixing of water between the inner and the outer Bays [25]. The inner Murchison Bay forms the main abstraction point for portable water supplied to the expansive population around Kampala city.

The major channels/wetlands that drain into the Murchison Bay include Nakivubo, which drains Kitante and Lugogo channels with inlets into the inner Murchison Bay; Kansanga wetland, which stretches into the Ggaba shoreline; Kinawataka, which drains industrial centres of Nakawa and Kyambogo; and Namanve wetland [28].

### 2.2. Spatial Extent and Distribution of Water Hyacinth

High-resolution satellite images covering the Murchison Bay were acquired from Sentinel-2 archives manned by the United States Geological Survey (USGS) (https://glovis.usgs.gov/web-link). The images were for the period between 2016 and 2019 with Sentinel-2 MSI tiles covering the study area. A single image was downloaded for each year and this had to be of the dry period (between January and March), during which there is less cloud cover to mask ground features. Images selected were those with less than 5% cloud cover as image analysis targeted the visible bands (RGB and IR). Sentinel-2 images were preferred to Landsat data due to the high spatial resolution of the former (Sentinel with bands in 20 *∗* 20 meters) compared to the latter (Landsat with 30 *∗* 30 meters). Since the launch of its first satellite in 2013, Sentinel data have become more and more applied in landscape mapping, thus serving as an alternative to coarse resolution Landsat series data [22]. The images were atmospherically corrected using the Dark Object Subtraction (DOSI) model under the semiautomated classification (SCP) embedded in Quantum GIS (QGIS) 3.12 software.

To determine the pattern and distribution of water hyacinth in the Murchison Bay, the preprocessed Sentinel-2A images were further processed using the maximum likelihood supervised classification algorithm in QGIS. The model distinguishes pixel properties for different land uses and cover (for which water hyacinth was part) based upon input training data of pixels representing the predefined land use/cover classes (Table 1). Based on these data, the algorithm groups the remaining pixels on an image into the created classes. The maximum likelihood classification model was selected for the satellite imagery classification in this study because of its high precision in land use and cover classification as reported in previous studies, e.g., [19, 22]. Moreover, Sentinel-2 data had never been applied in water hyacinth studies in the Murchison Bay.

In addition, field data collection was conducted to record the location of the water hyacinth using GPS (primary data) during November–December 2019 and January–February 2020. These were randomly generated sampling points across the Murchison Bay, following water hyacinth-infested areas. These points were used in a training data set for mapping the extent and pattern of water hyacinth.

The postprocessing of the classified Sentinel-2 images involved the computation of areal statistics for the cover classes for the images corresponding to the study period (2016 to 2019). Using discriminate analysis, the various changes in coverage of the water hyacinth vis-à-vis other covers in the Murchison Bay were determined, which indicated the pattern and distribution of the water hyacinth in the Bay over the study period. The results are presented in tables and graphs. The QGIS semiautomatic classification plug-in allows for the extraction of several classification accuracy statistics such as overall accuracies, user's accuracy, producer's accuracy, and kappa efficiency (Semi-Automatic Classification Plugin Documentation, release 5.3.2.1. 2017).

### 2.3. Perceived Determinants of Water Hyacinth Distribution

The study adopted a cross-sectional research design to establish determinants of water hyacinth extent and pattern in the Murchison Bay as perceived by the residents. The design followed a quantitative approach to gathering data from respondents using structured questionnaires. The targeted respondents' categories included officials from the Fisheries Department and National Water and Sewerage Corporation (NWSC) and traders and fishermen stationed at Port Bell, Ggaba, and Mulungu landing sites. A sample of 201 respondents from the abovementioned categories was drawn following purposive and stratified sampling techniques. First, the respondents' categories were defined on the criterion that they are involved in water resource management and are directly affected by water hyacinths and on the fact that they are more knowledgeable about the problematic waterweed (water hyacinth) in their areas of jurisdiction. Secondly, a stratified sampling technique was employed to select respondents from three landing sites around the Murchison Bay. Sixteen respondents (16) were selected from the Fisheries Departments at Ggaba, Mulungu, and Port Bell landing sites, respectively. One hundred and twenty (120) respondents were randomly selected from the three landing sites and 13 respondents from the National Water and Sewerage Corporation at Ggaba.

After determining the target respondents, semistructured questionnaire copies were hand delivered to collect participants' perceptions of the physical and human factors responsible for water hyacinth distribution in the Murchison Bay over the years. The main section of the questionnaire required respondents to rank the factors that they thought to influence the water hyacinth pattern and distribution in the Murchison Bay. Up to 15 factors were presented for ranking on a scale of 1–4 to show the extent to which a factor determines water hyacinth extent and distribution in the Bay (where 1 indicates the least level and 4 indicates the highest level of determination). Data obtained were computer coded in the Statistical Packages for Social Scientists (SPSS) computing program, version 23.0. The data were then analysed using both descriptive and inferential statistical techniques. Mean and standard deviation statistics were generated from the rated responses, and the results are presented in tables. Pearson's chi-square (*X*^2^) test was performed to establish whether the rated factors were related to water hyacinth spread and distribution across the Bay. The relationships were tested at alpha level 0.05. The analysis yielded Pearson's chi-square, likelihood, and *p* value statistics, which are also presented in tables.

### 2.4. Effects of Water Hyacinth on Physicochemical Water Quality Properties

**Figure 2:**
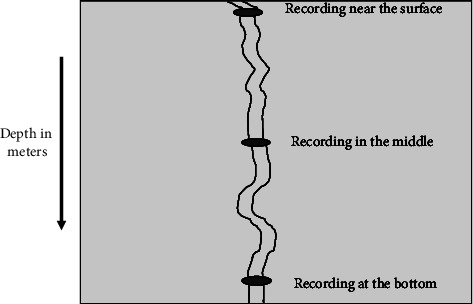
Sampling depth interval at each collection point.

**Figure 3:**
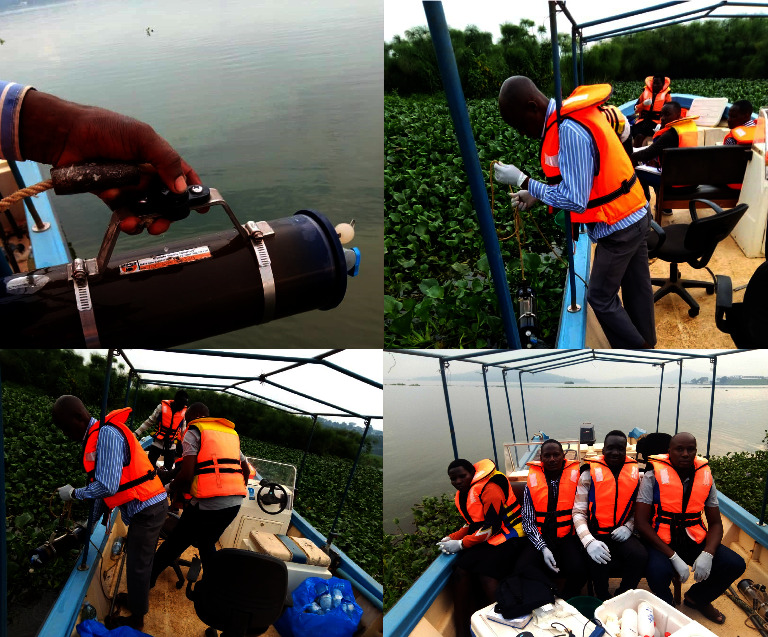
Water sample collection during the field study (source: authors).

For water physicochemical property analysis, water samples were collected from stationary floating water hyacinth areas and in water hyacinth-free environments (open lake). Ten pairs of sampling locations were determined, corresponding to the two environments. The sampling points had to be located at an average distance of 500 meters from one another. From each sampling environment, three samples were drawn in relation to water depth (i.e., near the water surface, middle, and at the bottom) (Figure 2). Water samples were then collected using a 1000 ml water sample collector and subsamples were poured into 500 ml plastic water sample bottles (Figure 3), which were stored in boxes before transportation to NWSC laboratories at Ggaba and Lubigi, for the analysis of specific water quality parameters of interest in this study. The bottles were washed with nitric acid to remove any form of contaminants and to ensure that the physical properties of the water samples were maintained. The parameters of interest included pH, water temperature, total phosphate (TP), dissolved oxygen (DO), biochemical oxygen demand (BOD), electrical conductivity (EC), chemical oxygen demand (COD), turbidity, and transparency. These were selected specifically because they are key indicators of overall water quality and thus impact human health, water production, and ecosystem health [3, 12, 32].

The sampling locations were accessed using a motorized boat, and at each sampling point, coordinates were recorded using Garmin Global Positioning Systems, whereas sampling in the open-water environment was done randomly and sampling in the water hyacinth environment was done purposively and systematically (following 500 m mean distance interval). Two different sampling occasions were conducted. The first sampling activity was conducted between September and December 2019. This period represented samples for the wet season of the study area climate zone. The second sampling activity was conducted between January and February 2020. This period represented sampling for the dry season. Thus, a combination of data from two different seasons accounted for any variations in water quality brought about by seasons (November/December 2019 and January/February 2020).

**Figure 4:**
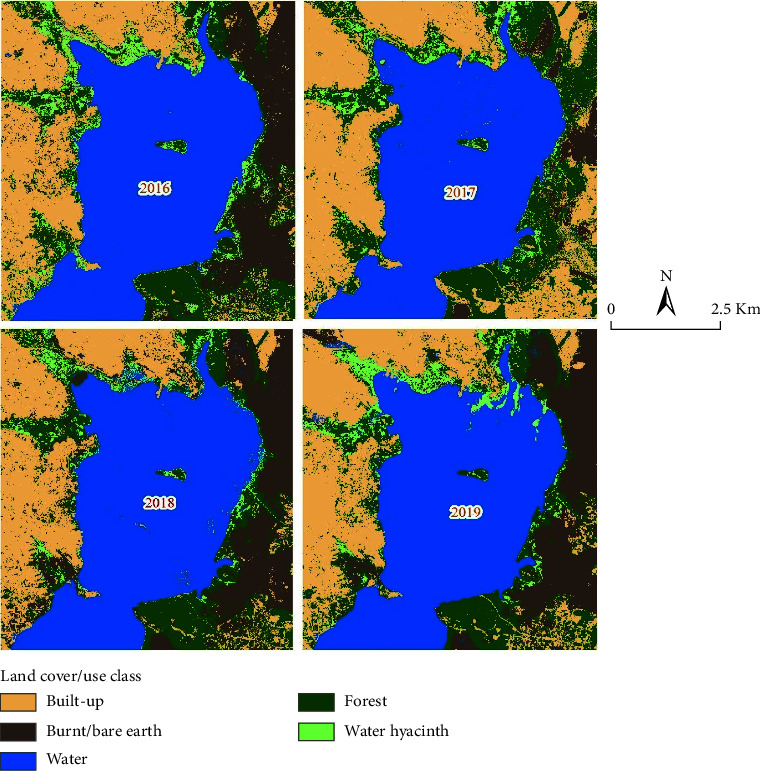
Water hyacinth relative extent in the Murchison Bay between 2016 and 2019.

DO, temperature, and transparency were tested and recorded in the field, while turbidity, pH, TP, EC, BOD, and COD were tested in the laboratory using set standard procedures [33, 34] (Figure 4). Temperature and DO were measured using a dissolved oxygen meter. The device was immersed in the collected water sample, and the results for both parameters were displayed on the device's digital screen. The temperature was recorded in degrees whilst DO was recorded in mg/L. Transparency on the other hand was measured using a Secchi disk, where the device was dipped into the water at every sampling point and the depth at which the disc was no longer visible was recorded in meters [35].

While in the laboratory, pH and EC were measured by the electrometry method using a pH/EC multimeter (Hach Sension + MM374). This device has two probes, one for measuring pH and the second for measuring conductivity. 100 ml of the sample was poured into a 100 ml beaker and the probes were lowered into the sample before starting the machine. The sample was stirred using a magnetic stirrer until a stable reading was obtained and displayed on the equipment display screen. The device displays both the pH and EC (mS/cm) values, which were recorded. pH has no units while EC was measured in mS/cm.

The turbidity of the water samples was determined using a turbid meter (Hach TL 2300). The sample was uniformly mixed and poured into a 40 ml cell up to the mark and then inserted into the machine to read off turbidity values in nephelometric turbidity units (NTU) displayed on the device's screen. COD determines the amount of oxygen required for the oxidation of organic matter using a strong chemical oxidant such as potassium dichromate under reflux conditions [36]. This test is widely used to determine the same types of pollution as the BOD expressed in milligrams per litre (mg/L). COD was determined by the oxidation of organic matter using acid dichromate solution, followed by spectrophotometric determination. The digestion tube and caps were washed with 4 ml H_2_SO_4_ to prevent contamination. Two ml of the sample was poured into the digestion tube, followed by adding 2.0 ml of potassium dichromate digestion solution. The abovementioned process allowed an acid layer to be formed under the sample digestion layer. Cap tubes were swirled several times to mix completely, without inverting the tubes. The solution was placed in a preheated oven of 150°C for 2 hrs. This was followed by reading the concentration of the sample with the help of a spectrophotometer DR 6000.

BOD measures the amount of oxygen consumed through the biochemical degradation of organic carbon, inorganic materials, and nitrogenous compounds present in waste water over a specified incubation period usually 5 or 7 days. It was determined by the preparation of dilution water by transferring a desired volume of water into a bottle and then saturating the water sample with DO by aerating with organic-free filtered air, adding 1 ml of each phosphate buffer, MgSO_4_, CaCl_2_, and FeCl_3_ solutions/l of saturated water, mixing thoroughly before starting to use, while the preparation of DO was conducted by adding the specific volume of the sample to the individual BOD bottles of known volume [37], filling the bottles up to the brim with sufficient dilution water, reading DO1 using the dissolved oxygen meter (Hach), then taking the initial reading, and tightly sealing the bottle leaving no air bubbles and incubating for 5 days at 20°C. After the 5-day incubation, residual DO was determined in the samples.

To determine total phosphates (in mg/L), organically combined phosphorus and all phosphates were converted to orthophosphate. To release the phosphorus as orthophosphate from organic matter, a wet oxidation technique was applied. This was based on wet oxidation with potassium per sulphate. The same procedure for orthophosphate determination was followed. The procedure involved the following: taking 25 ml diluted or whole samples, acidifying with 1 ml H_2_SO_4_, 0.04 M, adding 5 ml digestion reagent, mixing thoroughly and preparing blank (25 ml distilled water) and phosphate standard by taking 25 ml of known standard concentration, and treating both the blank and phosphate standards in the same way as the sample.

Physicochemical property data obtained using both field and laboratory methods were largely numeric and thus analysis involved the use of parametric statistical techniques. These data were organised in a Microsoft spread sheet and then imported into the R statistical computing environment. Using this program, first, exploratory and descriptive statistics were computed including maximum, minimum, 1^st^ quartile, median, 3^rd^ quartile, mean, variance, and standard deviation for each of the physicochemical water quality properties. These were computed for the two data sets representing water hyacinth and nonwater hyacinth environments, and the analysis was meant to summarize the data and give a snapshot of the emerging differences and similarities in the water quality parameters from the two sampling environments. In the second phase, data on the water quality parameters were subjected to two-way analysis of variance (ANOVA). That is, type III sums of squares [38] were computed on each of the water quality variables' data in relation to the sampling environment and water depth.

## 3. Results

### 3.1. Spatial Extent and Distribution of Water Hyacinth

**Figure 5:**
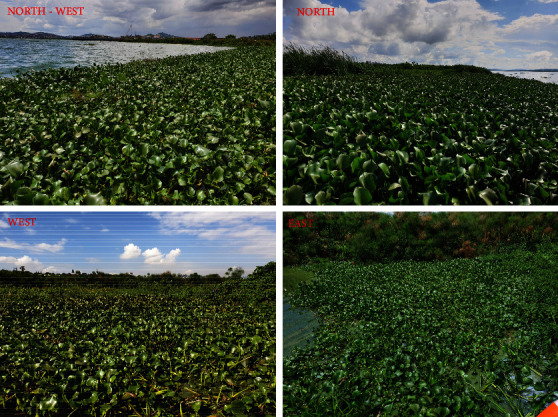
Distribution of the water hyacinth in the Murchison Bay (source: author, 2020).

Results from the satellite imagery classification indicate that, in 2016, water (42%) and built-up areas (24%) were the most predominant land use/cover types, followed by forest vegetation (15%) (Table 2 and Figures 4 and 5). Water hyacinth was the least cover type throughout the study period (2016 to 2019), which is 1% in 2016, 4% in 2017, 3% in 2018, and 4% in 2019, respectively. Between 2017 and 2019, water and built-up areas still dominated the land use/cover types, followed by forest vegetation compared to the areal extent for water hyacinth.

In all the studied periods, the built-up area increased by 1% in 2016–2017 and 2018–2019, but between 2017 and 2018, it declined by 2%. The areal extent of water and forest vegetation remained relatively static but experienced a 1% decline in 2019 (for the former) and in 2018 (for the later). On the other hand, water hyacinth experienced fluctuations during the study period, increasing between 2016–2017 and 2018–2019 but declining between 2017 and 2018. The results show that burning/bare ground was majorly responsible for the decline in vegetated areas (forest and water hyacinth) in 2018, followed by built-up areas majorly in 2016 (when water hyacinth had the smallest areal extent) while the decline in water body areal extent could be accounted for by the increase in the spread of the water hyacinth over the water body. From the analysis results, water hyacinth covered approximately 511 km^2^ in 2016, 2434 km^2^ in 2017, 1542 km^2^ in 2018, and 2138 km^2^ in 2019.

### 3.2. Perceived Determinants of Water Hyacinth Distribution

Results (in Table 3) reveal that water hyacinth extent and distribution on the lake were highly influenced by the sheltered morphology of the Bay, effluent discharge, strong winds, the speed of water currents, change in lake water level, construction activities at the shore, and ferry navigation (with average rating between 2.4 and 4) according to the respondents' opinions. However, water temperature, humidity, biotic colonization, hyacinth species, herbaria, water depth, fish hatcheries, and fishing gear received rating scores below 2.0 which on a scale of 1 to 4 is below average in terms of their importance in influencing water hyacinth distribution in the Murchison Bay. The results imply that the majority of the respondents believe that much of the water hyacinth proliferation is due to man's influence through sewage effluent discharge, construction works, and ferry navigation in the Bay.

From the chi-square results, the perception that water-level changes, strong winds, biotic colonization, humidity, construction at the shore, fishing boats and nets, herbaria, and ferry navigation determined water hyacinth extent and distribution positively and significantly (*p* < 0.05) varied spatially across the Murchison Bay. This implies that these factors significantly influenced water hyacinth spread and distribution, but in selected sections of the Bay, and thus not universally considered significant determinants. On the other hand, the perception that temperature, sheltered Bay, speed of water current, hyacinth species, water depth, and botanic gardens were important determinants was not significantly related to location difference in the Bay (*p* > 0.05). This result implies that water hyacinth spread and distribution in the Bay were equally perceived to be significantly determined by water temperature, sheltered morphology of the Bay, speed of the water, hyacinth species, water depth, and proximity to botanic gardens. Therefore, these factors are significant drivers of water hyacinth spread and distribution irrespective of the location in the Bay.

### 3.3. Effects of Water Hyacinth on Physicochemical Water Quality Properties

Analysis of water physicochemical properties revealed higher pH in the open lake environments with values ranging between 7.3 and 10.8 as compared to that in water hyacinth environments (Table 4). The water pH differed significantly between sampling sites and lake depth (Table 5) which means that water hyacinth and water depth significantly (*p* < 0.05) affected water pH in the Murchison Bay. However, the interactive effect of these two variables was not significant, implying that the two variables affected water quality independently. Electrical conductivity average values in both water hyacinth-infested areas and open lake sites differed slightly (Table 4). However, the results indicated that water depth significantly affected the electrical conductivity of water. The ANOVA results for the interaction of the two factors (environment and lake depth) revealed no statistically significant effect of these variables on water EC (*p* > 0.05) (Table 5). The results signify that water EC in the Murchison Bay was significantly altered by water depth rather than by water hyacinth infestation.

The results also showed that water temperature in water hyacinth environments was slightly higher (27°C) than that in open lake water (26°C) on average (Table 4). However, ANOVA results indicated that although temperature varied between the water hyacinth sites, open lake, and lake depth, the differences were not statistically significant (*p* > 0.05) (Table 5). In addition, none of the interactions between the three factors had a statistically significant effect on water temperature. Descriptive statistics further revealed differences in the DO in the three sampling environment categories. However, water hyacinth-infested sites registered lower DO compared to an open lake environment (7 mg/L vs. 9 mg/L). The variations in the DO were significant for independent measurements related to lake depth and sampling environment but not statistically significant for the combined variables. This implies that water hyacinth deprived infested environments' water of DO.

Higher turbidity was also reported in water hyacinth-infested areas as compared to open lake sites. The effect of lake depth on the other hand was not significant on turbidity (*p* > 0.05). Additionally, the interactive effect of the sampling environments and lake depth on turbidity was also insignificant. The results relate to the fact that the water hyacinth negatively contributed to water turbidity in the Murchison Bay. Similarly, in terms of transparency, water hyacinth-infested areas were less transparent as compared to open lake sites. In addition, the effect of lake depth, as well as the interactive effect of the two factors (environment and depth), was not statistically significant (*p* > 0.05) (Table 5). This means that the water hyacinth increased the concentration of suspended materials in water which lowered transparency in the Murchison Bay.

The effect of the water sampling environment on total phosphates was statistically significant (*p* < 0.05). However, the effect of water depth and the interactive effect of the three factors on TP were not significant. Further, the effects of sampling environment and depth on the concentration of TP were independent of each other. This result suggests that water hyacinth significantly affected the concentration of total phosphates in the Murchison Bay. In addition, values of both BOD and COD were higher under the water hyacinth sites (Table 4) and the effect of the two variables was statistically significant, but the effect of lake depth was not significant (*p* > 0.05) accounting for the variations in COD. The interactive effect of the three factors was also not significant. This means that water hyacinth can account for an increase in BOD and COD in the Murchison Bay.

## 4. Discussion

### 4.1. Spatial Extent and Distribution of Water Hyacinth

The extent and pattern of distribution of water hyacinth varied largely over space and time. This study reveals that the increase in water hyacinth extent over the Murchison Bay is related to the findings in a study in [39] while assessing changes in water hyacinth coverage over water bodies in the northern Bangalore using Indian Remote Sensing Satellite LISS-II and III images of the years 1988–2001. Their study indicated that the area under water hyacinth increased in the recent years which consequently reduced the area under open water. The major areas of contention in the current study findings with those of [39] are due to the fact that water hyacinth coverage changes alternated between increases and decreases over the years. The present study established that the water hyacinth was mainly concentrated in the northern parts of the Murchison Bay. This revelation is also echoed in [40] who reported that the water hyacinth attained a maximum lake-wide extent of approximately 17,374 ha by 1998 on the northern shores of Lake Victoria to which the Murchison Bay belongs. This points towards the area of intervention in terms of control of the waterweeds. The results also indicate that water hyacinth coverage largely increased with a decrease in water surface area, which means that water hyacinth reduces the exposed water surface for other environmental processes such as atmospheric water transfer.

### 4.2. Perceived Determinants of Water Hyacinth Distribution

This study established that the sheltered morphology of the Bay, effluent discharge (sewage), strong winds, the speed of the water currents, water-level changes, construction activities at the shore, and ferry navigation strongly determined the water hyacinth pattern and distribution in the Murchison Bay as perceived by the respondents although with variations in the level of influence. This revelation is directly implied in the report in [21] that currents constitute the dispersion of water hyacinth propagule and stolon which makes the weed get distributed and colonize new areas within a short time. The speed of water currents is thus an abiotic factor for the colonization of new areas with considerable importance for the potential propagation of the infestation in a given territory. However, temperature and humidity insignificantly influenced water hyacinth extent and distribution in the Murchison Bay. The overall temperatures and humidity over the Bay are however generally high (above 18°C and 70%, respectively) on average. The current study also indicated that the influence of water depth on water hyacinth extent and distribution is minimal as perceived by the respondents in the Murchison Bay catchment. However, previous studies [13] have shown that both the depth of the water and changes in lake water levels are important for the growth and expansion of water hyacinths. The reports suggest that the plants have more roots in deep waters than in shallow waters, while the leaf area and the summer growth of the plant are greater in shallow waters [41]. This implies that whereas people in the Murchison Bay thought that water depth plays an insignificant role in water hyacinth distribution and extent, the factor is crucial as even the results from mapping showed more concentration of the water hyacinth on the shores of the lake where lake depth significantly reduces.

### 4.3. Effects of Water Hyacinth on Physicochemical Water Quality Properties

This study further assessed the effect of water hyacinth on water quality, and from the analysis, water hyacinth altered the aquatic environment just as was reported in [14] leading to increased water temperature, concentrations of turbidity, COD, and BOD. Temperature is a major determinant of many chemical reactions that take place in water [32]. Warmer temperatures (>25°C) hold less dissolved oxygen which is key for the survival of aquatic organisms but also increase the solubility and consequent toxicity of compounds such as zinc and lead [1]. Average water temperatures of 25–27°C recorded in this study are within the permissible limits of the World Health Organization (30°C) as well as those reported in similar studies [42]. Although different, the temperature variation in the two environments was not significant. The higher temperature values under the water hyacinth could be attributed to the heat generated from the breakdown of organic matter, below the hyacinth [13]. In addition, the location of the Bay in the equatorial belt could be the reason for the small and insignificant variation in temperature reported.

The water hyacinth was noted to lower dissolved oxygen. Dense water hyacinth mats not only interfere with free oxygen transfer between the water and the atmosphere but also limit the mixing of the water by wind, leading to lower levels of dissolved oxygen [14]. While high DO levels add taste to water, it also has a highly corrosive effect on water pipes when at extreme values [42]. Biological processes related to plant decomposition can lead to a reduction in the concentrations of DO [43], which could explain the lower DO levels in the water hyacinth environment. Concentrations between 5 and 10 mg·L^−1^ are ideal for the proper functioning of aquatic systems, which gives validity to the ranges reported in this study. Dissolved oxygen is dependent on water temperature and the biological demand of the lake system [3]. Temperature affects the ability of water to dissolve oxygen to solubility at different temperatures, in that, a lower temperature improves the dissolution of oxygen compared to a higher temperature. In the current study, higher temperatures were recorded under water hyacinth-infested areas which are accounted for by the fact that decomposing water hyacinth releases heat that warms the water and this reduces the dissolved oxygen [44].

Water hyacinth also increased the acidity of water in the Murchison Bay. The lower pH values in areas infested by the water hyacinth could be attributed to the accumulation of carbon dioxide below the weed [20]. pH extremes negatively impact water quality as well as determine coagulants used in water purification and treatment [45]. pH values of more than 7 have been reported to change the taste of water and make its treatment more costly [46] while lower pH values (less than 5) are associated with the corrosion of metals due to the higher levels of acidity [47]. This damages metallic water distribution infrastructure such as pipes in addition to contamination of the water being distributed. Reference [48] reports that water pH determines the solubility (the amount that can dissolve in water) biological availability (the amount that can be utilised by aquatic life for chemical constituents such as nutrients (phosphorus, nitrogen, and carbons) and heavy metals (lead, copper, and cadmium). The pH values reported in this study, although slightly different, were in optimal ranges within the permissible ranges by WHO standards between 6.5 and 8.5 [8].

Water hyacinth also increased turbidity levels of the lake water in the study area. The biological and chemical reactions in water hyacinth-infested areas increase constituents within the water, which increases turbidity levels [49]. The increased materials not only increase the costs of disinfection but also increase the risks of inhabitation by pathogenic organisms, which all complicate the process of water production as more chemicals are required for disinfection and coagulation [50]. Suspended materials also lead to the clogging of filters leading to increased run-time [51]. Suspended materials further attract metals such as lead, mercury, and chromium, as well as other organic pollutants, which deteriorate the quality of water. The materials also lower transparency as they limit the amount of light transmissible through the water. This is consistent with the findings in [15] that water clarity can be greatly modified by the waterweed as well as the reduced concentration of nutrients such as nitrogen and phosphorus. Decreased transparency has also been associated with a breakout in algae bloom because of increased photosynthesis rates, hence requiring more chlorine during peroxidation. The mean turbidity value obtained for this study is very high when compared with the WHO-recommended value of 5.00 NTU, and this can lead to an increased demand for chlorine during disinfection [51].

Findings revealed low electric conductivity of water under the water hyacinth-covered lake areas. The low EC values (142.3 mS/cm) are indicative of relatively good water quality and an indicator of low total dissolved solids (TDS) as has been reported in related studies like [42, 52]. The significance of electrical conductivity is in its proportion of saltiness, which enormously influences the water taste and, in this manner, significantly affects the convenience of water. Besides, it is associated with high levels of corrosiveness, which is also detrimental to the metallic water production infrastructure. The WHO standards provide for an EC value of not more than 400 mS/cm [53].

Although the BOD and COD levels in the water hyacinth environment were slightly lower, the variation in open water was insignificant. Increasing values of COD are often caused by increased organic matter in the water [54]. Similarly, water with a high COD has a high chlorine demand and requires high chlorine dozes to fully disinfect [27]. This is also because high COD values are associated with increased organic pollution in the water [29]. The more organic material there is in the water, the higher the BOD used by the microbes will be [1]. The fact that BOD was higher in underwater hyacinth environments is proof that the water hyacinth increases the need for oxygen in water [46]. Water with high BOD requires increased use of coagulants to achieve effective clarification, thus an important indicator of overall water quality. While unpolluted water has BOD values of less than 5 mg/L, values up to 32.85 mg/L as reported in this study show some level of pollution in the water requiring a higher level of treatment.

While some studies have demonstrated the potential use of the water hyacinth and other aquatic plants in reducing suspended solids, dissolved solids, electrical conductivity, hardness, biochemical oxygen demand, chemical oxygen demand, dissolved oxygen, nitrogen, phosphorus, heavy metals, and other contaminants [41, 54, 55], the findings from the present study indicate otherwise. In [13], water hyacinth is reported to have significantly increased water conductivity and total dissolved solids. However, these contradictions could be explained by the fact that the water hyacinth performs the purification functions only under controlled/managed conditions but for the alien infestations in the Murchison Bay in Lake Victoria and other freshwater lake systems in the region, it is possible that water hyacinth instead uncontrollably creates conditions for deterioration of water quality. Therefore, there is a need to manage the water hyacinth for water purification under controlled conditions and also convert it to other uses such as farm mulch and biogas generation as suggested in [54, 56].

## 5. Conclusion

The extent and distribution of the water hyacinth in the Murchison Bay vary over space and time but are more concentrated on the northern shores. The results revealed shifts and differences in area coverage of water hyacinth in the Bay over the four years (2016–2019). Water hyacinth significantly affects water quality, in some cases, outside the WHO maximum-minimum permissible limits. Results from this study indicate that parameters such as DO, turbidity, pH, BO, and total phosphates are not within the permissible range of the WHO 2020 guidelines. The effect of sampling depth was only significant on pH, EC, BOD, and DO whilst the interactive effect of environment and depth was insignificant for all water quality parameters. The determining factors of water hyacinth extent and distribution pattern largely vary over space. The water hyacinth determinants include strong winds, herbaria, fishing gear, construction activities at the shore, water-level changes, fish hatcheries, ferry navigation, humidity, and biotic colonization.

## Figures and Tables

**Table 1:** The land cover/use types' classification system used for the Murchison Bay area.

Land cover/use class	Description
Built-up/settlements	Land consisting of residential areas, commercial buildings, and slums and associated infrastructure such as roads
Burnt/bare earth	Areas with burnt vegetation and/or exposed earth as a result of vegetation removal
Lake	Areas covered by lake water in the Bay
Forest	Areas under naturally existing and/or planted tree cover
Water hyacinth	Areas covered by the water weed and host wetland vegetation within the water body

**Table 2:** Areal extent statistics of land cover around the Murchison Bay between 2016 and 2019.

Year	Built-up(km^2^)	(%)	Burnt/bare earth (km^2^)	(%)	Water (km^2^)	(%)	Forest (km^2^)	(%)	Water hyacinth (km^2^)	(%)
2016	12006	24	8746	18	21414	42	7287	15	511	1
2017	13058	25	7108	14	21985	42	7663	15	2434	4
2018	12025	23	9461	18	21807	42	7414	14	1542	3
2019	12715	24	8282	16	21517	41	7596	15	2138	4

**Table 3:** Mean rating, standard deviation, and Pearson's chi-square results showing the relationship between the water hyacinth determinants and landing site.

Factors	Mean rating	Standard deviation	Pearson value	Likelihood ratio	*p* value
Morphology of the Bay	3.2	0.9	4.6	4.4	0.598
Strong winds	3.2	1.1	39.4	39.6	≤0.001
Water level changes	2.7	1.9	20.7	20.3	0.002
Speed of water currents	3.0	1.9	4.3	4.2	0.633
Hyacinth species genetics	1.4	0.7	4.7	4.4	0.580
Water depth	1.5	0.8	14.1	16.0	0.29
Biotic colonization	1.3	0.6	12.6	16.4	0.049
Temperature	1.2	0.4	0.8	1.2	0.935
Humidity	1.2	0.5	15.7	17.5	0.015
Discharge of effluent	3.2	0.9	13.5	11.3	0.35
Construction at the shore	2.5	1.1	23.7	24.5	≤0.001
Fish hatcheries	1.8	0.2	17.8	18.7	0.007
Proximity to botanic gardens	1.9	1.0	8.3	8.5	0.216
Fishing boats and nets	1.8	1.1	33.1	40.3	≤0.001
Herbaria	1.5	0.9	29.1	28.1	≤0.001
Ferry navigation	2.5	1.2	19.5	23.4	0.012

**Table 4:** Summary statistics for water quality parameters in the open lake and hyacinth-infested areas.

Water hyacinth environment statistics	pH	EC (mS/cm)	Turbidity (NTU)	COD (mg/L)	TP (mg/L)	BOD (mg/L)	Temp (OC)	DO (mg/L)	Transparency (meters)
No. of observations	120	120	120	120	120	120	120	120	120
Minimum	5.5	102.1	6.05	16.0	0.2	17	24.1	5.3	0.1
Maximum	7.6	313	544	155	8.3	48	42	9	1
1st quartile	6.2	122	54.4	56	2.5	28	25	6.5	0.3
Median	6.7	132.9	59.6	70	3.2	35	25.4	7.0	0.4
3rd quartile	7.1	160.2	63.3	96.8	3.3	38.1	26.3	7.4	0.5
Mean	6.7	146.8	66.3	77.2	3.7	32.9	27	6.9	0.4
Variance	0.3	1870.8	3643.3	867	1.6	54	20	0.5	0.1
Standard deviation (*n*-1)	0.6	43.3	60.4	29.4	1.2	7.3	4.5	0.7	0.3

Open lake environment statistics	pH	EC (mS/cm)	Turbidity (NTU)	COD (mg/L)	TP (mg/L)	BOD (mg/L)	Temp (OC)	DO (mg/L)	Transparency (meters)

Minimum	7.3	99.7	29.1	17	0.1	3.0	0.10	6.7	0.1
Maximum	10.8	408	59	211	4.1	30	41	10.9	1.1
1st quartile	7.9	125.2	45.6	51	1.1	19.2	24.7	8.0	0.4
Median	8.4	132.3	48.3	55.5	1.4	23.2	25.2	8.5	0.6
3rd quartile	8.7	153.3	52.8	63.8	1.8	25.7	26	9.2	0.9
Mean	8.5	142.3	48.1	62.9	1.4	21.8	25.7	8.6	0.6
Variance	0.6	1752.5	38.2	909.2	0.6	39	26.6	0.9	0.1
Standard deviation (*n*-1)	0.8	41.9	6.2	30.2	0.8	6.2	5.2	0.9	0.3

**Table 5:** Analysis of variance for physicochemical water properties: type III sums of squares.

Main effects	pH	EC (mS/cm)	Turbidity (NTU)	COD (mg/L)	TP (mg/L)	BOD (mg/L)	Temp (OC)	DO (mg/L)	Transparency (meters)
A: environment									
Sum of squares	101.1	600.0	9542.0	6037.0	108205.0	3714.2	42.0	84.1	0.4
Df	1.0	1.0	1.0	1.0	1.0	1.0	1.0	1.0	1.0
Mean squares	101.1	600.2	9542	6038	108.2	3714.2	42.0	84.1	0.4
F-ratio	238	0.3	4.6	7.1	1001	98.3	1.8	144	5.1
*p* value	<2*e* − 16^*∗∗∗*^	0.7	0.03^*∗*^	0.009^*∗∗*^	<2*e* − 16^*∗∗∗*^	<2.2*e* − 16^*∗∗∗*^	0.2	<2.2*e* − 16^*∗∗∗*^	0.03^*∗*^
B: depth									
Sum of squares	2.6	11699.0	1132.0	2298.0	5.7	1099.8	29.9	14.5	0.0
Df	2.0	2.0	2.0	2.0	2.0	2.0	2.0	2.0	2.0
Mean squares	1.3	5849.3	566	1149.1	2.8	549.9	14.9	7.2	0.0
F-ratio	3.1	3.3	0.3	1.3	2.6	14.5	0.6	12.4	0.0
*p* value	0.04^*∗*^	0.04^*∗*^	0.8	0.3	0.1	2.361*e* − 06^*∗∗∗*^	0.5	1.371*e* − 05^*∗∗∗*^	1.0
A^*∗*^B (interaction)									
Sum of squares	0.5	514	2794	4674	3.5	166.2	11.3	0.2	0.0
Df	2	2	2	2	2	2	2	2	2.0
Mean squares	0.2	257.2	1397	2337	122.5	83.1	5.7	0.1	0.0
F-ratio	0.6	0.1	0.7	2.7	144.0	2.2	0.2	0.2	0.0
*p* value	0.6	0.9	0.5	0.1	1.1	0.1	0.8	0.8	1.0
Residual	49	202268	234909	97537	122.5	4309.3	2662	530	3.0
Total (corrected)	153	216	248377	110546	1224	9290	2536.2	629.2	3.4

^*∗*^, ^*∗∗*^, and ^*∗∗∗*^ = statistically significant at 95%, 99%, and 100%, respectively.

## Data Availability

The data used to support the findings of this study are available from the corresponding author upon request.
